# On the impact of nonresponse in logistic regression: application to the 45 and Up study

**DOI:** 10.1186/s12874-017-0355-z

**Published:** 2017-05-08

**Authors:** Joanna J. J. Wang, Mark Bartlett, Louise Ryan

**Affiliations:** 1 School of Mathematical and Physical Sciences, University of Technology Sydney, Ultimo, Australia; 2 The Sax Institute, Sydney, Australia; 3 The Australian Research Council Centre of Excellence for Mathematical and Statistical Frontiers (ACEMS), Parkville, Australia; 4 Department of Biostatistics, Harvard T.H. Chan School of Public Health, Boston, USA

**Keywords:** 45 and Up Study, Nonignorable missing, Nonresponse, Bayesian selection model, Sensitivity analysis

## Abstract

**Background:**

In longitudinal studies, nonresponse to follow-up surveys poses a major threat to validity, interpretability and generalisation of results. The problem of nonresponse is further complicated by the possibility that nonresponse may depend on the outcome of interest. We identified sociodemographic, general health and wellbeing characteristics associated with nonresponse to the follow-up questionnaire and assessed the extent and effect of nonresponse on statistical inference in a large-scale population cohort study.

**Methods:**

We obtained the data from the baseline and first wave of the follow-up survey of the 45 and Up Study. Of those who were invited to participate in the follow-up survey, 65.2% responded. Logistic regression model was used to identify baseline characteristics associated with follow-up response. A Bayesian selection model approach with sensitivity analysis was implemented to model nonignorable nonresponse.

**Results:**

Characteristics associated with a higher likelihood of responding to the follow-up survey include female gender, age categories 55–74, high educational qualification, married/de facto, worked part or partially or fully retired and higher household income. Parameter estimates and conclusions are generally consistent across different assumptions on the missing data mechanism. However, we observed some sensitivity for variables that are strong predictors for both the outcome and nonresponse.

**Conclusions:**

Results indicated in the context of the binary outcome under study, nonresponse did not result in substantial bias and did not alter the interpretation of results in general. Conclusions were still largely robust under nonignorable missing data mechanism. Use of a Bayesian selection model is recommended as a useful strategy for assessing potential sensitivity of results to missing data.

**Electronic supplementary material:**

The online version of this article (doi:10.1186/s12874-017-0355-z) contains supplementary material, which is available to authorized users.

## Background

Handling missing data and non-response represents one of the most methodologically challenging aspects of longitudinal survey research. The loss of cohort members over time can arise from failure to locate or contact them, or because members refuse to participate for various reasons. Missing data and nonresponse constitute problems for epidemiological studies for two main reasons. First, missingness leads to the loss of observations and the reduction of sample size. This can result in a loss of statistical power and an increase in variances of estimates. The second consequence of nonresponse is that estimates may become biased, because the decision to respond to a survey is rarely completely random. Those members who responded to the follow-up surveys may have different characteristics from the nonresponders. In fact, many studies have found nonresponse is commonly associated with demographics, socioeconomic status and health behaviours and conditions ([1–3]). Hence, the responders may not be representative of the original sample and estimated measures of associations between exposure and outcome based solely on responders can be biased ([4, 5]).

It is possible to adjust and accommodate for missing responses. To compensate for the loss of participants in longitudinal surveys, it is common to assign weights, usually derived from the probability of response, to the responders to ensure the distribution of the original population is properly represented by the responders [6]. A second commonly used approach is multiple imputation, where the missing values are imputed based on statistical models which produce estimated plausible values [7]. Studies that use these methods to account for nonresponse have not found serious bias in association estimates ([8–11]). However, results from analyses based on weighting and multiple imputation methods are generally valid under the assumption that the data are “missing at random” (MAR), which means that missingness depends only on the observed data. Apart from multiple imputation, another commonly used approach is full information maximum likelihood (FIML) method which estimates parameters directly using all the information contained in the incomplete data set by maximising the observed data likelihood. Another popular maximum-likelihood based missing data method is the expectation-maximisation (EM) algorithm, which estimates the parameters directly by iterating between the E step and the M step. Both FIML and EM assume MAR mechanism and multivariate normality for the joint distribution of all variables.

In many situations there may be a reason to believe that even after accounting for the observed information, responders still differ from non-responders. In other words, the process that generates missingness may be directly related to the values of the unobserved variables. For example, people with very low or very high incomes may choose not to reveal their salaries. In the longitudinal study that motivates this paper, it is reasonable to think that people who move to a new residence during the follow-up period may be less likely to respond to the follow-up survey. Such cases, assuming that the data are MAR, may yield biased results. A number of authors in recent years have discussed strategies to handle this so called informative missingness. For instance, Diggle et al. [12] proposed a selection model for continuous longitudinal data with informative drop-out, that combines a linear model for the outcome and a logistic regression model for the drop-out process. Such selection models have also been explored by Scharfstein et al., Ibrahim et al. and Carpenter et al. [13–15], among many others. On the other hand, the pattern-mixture approach [16] models the distribution of data conditional on the missing data pattern. Applications of the pattern-mixture model include [17–19]. A comprehensive review and discussion of these models can be found in [20–23]. Recently, Wang et al. [24] proposed a Bayesian sensitivity analysis to address the problem of missing response data in the context of logistic regression based on a longitudinal follow-up study. They also showed how to quantify the likely impact of bias associated with informative missingness when naïve methods are used. In this paper, we illustrate the use of this methodology in the context of the 45 and Up Study, using our results to make recommendations for broader epidemiological practice.

The 45 and Up Study is a large-scale Australian cohort study of individuals aged 45 and over. Recruitment into the 45 and Up Study commenced in early 2006 and the first 45 and Up Study follow-up survey was administered in 2012. The cohort consists of more than 267,000 men and women aged 45 years and over from the general population of the state of New South Wales [25]. Extensive information was collected at baseline on demographic and social characteristics; personal health behaviours; general health related data such as known risk factors for major causes of morbidity and mortality and other likely confounding factors. The 45 and Up Study aims to provide researchers with reliable information on a wide range of exposures and outcomes of public health for informing policy to support healthy aging.

A rigorous evaluation of possible impact of nonresponse in the 45 and Up Study has not been attempted. This study aims to fill this research gap by identifying sociodemographic, general health and wellbeing characteristics associated with nonresponse to the follow-up questionnaire and assessing the extent and effect of nonresponse on statistical inference drawn from estimates based on the 45 and Up Study survey data. In particular, we allowed for the possibility that nonresponse was non-ignorable and we illustrate the use of Bayesian selection model approach [24] which allowed us to examine the sensitivity of our conclusions to different assumptions on the missing data mechanism. The results of this study provide insights into factors associated with nonresponse and methods that are useful for exploring and mitigating the consequences of nonresponse in the 45 and Up Study.

## Methods

### The 45 and Up study

The Sax Institute’s 45 and Up Study is a population-based sample from the state of New South Wales (NSW), Australia. Extensive demographic and social characteristics, personal health behaviour and general health-related data on individuals are collected. This provides researchers with reliable information on a wide range of exposures and outcomes of public health. The 45 and Up Study as a research resource will also give government the tools for evidence-based policy making to support healthy ageing.

Prospective participants were randomly sampled from the Department of Human Services (formerly Medicare Australia) enrolment database which provides a near complete coverage of the population. The study oversampled individuals from rural areas and those aged 80 years and over. Participants consented to regular follow-up and linkage of their survey data to a range of health databases. Recruitment commenced in February 2006 and the full cohort of size 267,157 reached by December 2009. The response rate to the 45 and Up Study is about 18% and participants included about 11% of the NSW population aged 45 years and over. Detailed description of the 45 and Up Study can be found in [25].

The first follow-up of participants began in 2012 with 41,440 45 and Up Study participants invited to respond. Of these, 27,036 returned the follow-up questionnaire, resulting in a response rate of 65.2%. After excluding individuals with missing values for baseline covariates, 32,037 individuals were included in this analysis with 21,750 of these being responders to the follow-up questionnaire.

### Data collection and variables

The 45 and Up Study baseline and follow-up questionnaires include demographic data such as age, postcode of residence, education, country of birth and type of housing, lifestyle factors including physical functional capacity, self-rated health condition and social support and marital status, employment status and household income. To explore the impact of nonresponse on measures of association, we focus on an outcome related to dwelling-type change between baseline and the follow-up. The 45 and Up Study questionnaires ask respondents to describe their dwelling type as belonging to one of eight categories: house, flat/unit/apartment, house on farm, retirement village/self-care unit, nursing home, hostel for the aged, mobile home and other. Due to low counts in some categories of these variables, house and house on farm; retirement village, nursing home and hostel for the aged; mobile and other are combined. Similarly, outer regional, remote and very remote Accessibility/Remoteness Index of Australia (ARIA) categories are combined. Physical functional limitation was assessed using the RAND 36-Item Health Survey, Version 1.0, subscale. The subscale was scored as recommended in ‘Scoring Instructions for MOS 36-Item Short Form Survey Instrument (SF-36) [26]. Social connectedness was assessed using the Duke Social Support Index (DSSI) subscale and scored as recommended by [27]. As per Phongsavan et al. [28], due to the positively skewed distribution of the social connectedness scores, this variable was transformed into quartiles.

In this paper, the outcome of interest was change in dwelling type between surveys, which was assessed by comparing responses to the relevant questions between the baseline and the follow-up surveys. To gain a better understanding of the particular type of housing transition, we focus on the case where the binary outcome represents transition into retirement village/nursing home/hostel for the aged, limited to those 45 and Up participants who were not in these categories at baseline. For ease of exposition, we subsequently refer to the outcome of interest as “transition to residential aged care”, or sometimes simply “transition”. It is important to explore various demographics, socio-economic and health factors associated with this transition as the findings of this study provide useful insights into relocation behaviour as people age and implications for aging and housing policy and age care provision.

### Statistical analyses

We will consider two statistical methods to adjust for nonresponse: inverse probability weighting using a propensity score and a Bayesian selection model. Both of these methods require a formulation of the model which predicts the probability of responding, given a set of observed covariates. As discussed below, the Bayesian selection model also allows for the possibility that missingness might depend on the unobserved response variable. We first conduct univariate chi-squared tests of association, to identify significant differences between responders and nonresponders in terms of demographic characteristics (age, gender, education qualification, country of birth, area remoteness), wellbeing (self-rated health, level of mobility, social support), household income, carer status, marital status and dwelling type at baseline.

The propensity score, as defined in Little [29], is the conditional probability that an individual responds, given a set of covariates. Following convention, we use a multivariable logistic regression model with response to the follow-up as the outcome variable to estimate propensity scores. Those variables with significant univariate associations with response status were included in a multivariable logistic regression. Variables with *p*-values > 0.05 were removed from the model in a stepwise fashion. The likelihood ratio test and model comparison using Akaike’s Information Criterion were used for variable selection. The final propensity score model comprises of the variables included in Table 1. These also formed the basis of the modelling for the nonresponse probability in the selection model described below.

**Table 1:** Characteristics of 45 and Up participants according to response to follow-up survey

Baseline characteristics	Moved *n* = 347 (%) (1.7%)	Responded *n* = 20730 (%) (67.3%)	Total *n* = 30799	OR (95% CI)
Gender				
Male	157 (1.7)	9232 (66.5)	13888	Ref
Female	190 (1.7)	11498 (68.0)	16911	1.11 (1.05–1.17)
Age (yrs)				
45–54	6 (0.1)	6577 (66.0)	9960	Ref
55–64	59 (0.7)	8696 (70.4)	12350	1.22 (1.15–1.30)
65–74	132 (3.4)	3920 (67.5)	5805	1.12 (1.02–1.23)
75+	150 (9.8)	1537 (57.3)	2684	0.81 (0.72–0.91)
Highest qualification				
None	34 (2.4)	1421(52.1)	2725	Ref
Year 10	82 (2.1)	3950 (62.8)	6294	1.25 (1.14–1.37)
Year 12	29 (1.4)	2019 (63.7)	3171	1.45 (1.30–1.62)
Trade	35 (1.9)	1855 (62.7)	2957	1.34 (1.20–1.50)
Cert./diploma	86 (1.7)	5042 (70.8)	7125	1.78 (1.62–1.96)
Tertiary	81 (1.3)	6443 (75.6)	8527	2.29 (2.07–2.53)
Area remoteness				
Major cities	192 (1.8)	10885 (66.1)	16460	Ref
Inner regional	135 (1.7)	7831(69.0)	11352	1.06 (1.00–1.12)
Outer regional/remote/very remote	20 (1.0)	2014 (67.4)	2987	1.06 (0.97–1.16)
Country of birth				
Australia	261 (1.6)	16237 (69.1)	23492	Ref
NW Europe	65 (2.5)	2652 (68.8)	3857	0.97 (0.89–1.04)
S & E Europe	5 (1.4)	356 (48.8)	730	0.67 (0.56–0.79)
Middle East	0 (0.0)	106 (42.6)	249	0.50 (0.38–0.66)
SE Asia	4 (2.0)	204 (44.9)	454	0.47 (0.38–0.58)
NE Asia	0 (0.0)	168 (46.9)	358	0.56 (0.44–0.70)
S & Central Asia	0 (0.0)	102 (50.2)	203	0.50 (0.37–0.67)
America	1 (0.4)	223 (60.4)	369	0.62 (0.50–0.78)
Sub Saharan Africa	4 (2.3)	177 (58.8)	301	0.57 (0.45–0.72)
Oceania	7 (1.4)	491 (64.0)	767	0.76 (0.65–0.89)
Speak a language other than English at home				
No	329 (1.7)	19453 (68.9)	28234	Ref
Yes	18 (1.4)	1277 (49.8)	2565	0.68(0.61–0.76)
Marital status				
Single	19 (1.9)	1013 (63.2)	1604	Ref
Married/de facto	252 (1.5)	16368 (68.5)	23908	1.13 (1.01–1.27)
Widowed/divorced/separated	76 (2.3)	3349 (63.3)	5287	1.01 (0.89–1.14)
Work status				
FT/self-employed	21 (0.2)	8996 (68.5)	13126	Ref
PT	20 (0.6)	3148 (71.2)	4422	1.18 (1.08–1.27)
Fully retired	276 (4.5)	6162 (66.7)	9243	1.34 (1.23–1.46)
Partially retired	13 (1.5)	865 (74.3)	1164	1.35 (1.17–1.55)
Disabled/sick	3 (0.7)	442 (51.3)	862	1.07 (0.91–1.26)
Unemployed/look after home/study/unpaid	14 (1.3)	1117 (56.4)	1982	0.86 (0.77–0.96)
Income category				
< $20,000	101 (3.4)	2951 (58.7)	5027	Ref
$20,000–$40,000	97 (2.6)	3696 (67.1)	5512	1.11 (1.02–1.21)
$40,000–$70,000	61 (1.3)	4598 (70.6)	6517	1.23 (1.12–1.34)
> $70,000	34 (0.5)	6890 (73.3)	9394	1.25 (1.13–1.38)
Prefer not to answer	54 (2.1)	2595 (59.7)	4349	0.84 (0.77–0.92)
Dwelling type				
House/house on farm	272 (1.5)	18419 (68.0)	27086	Ref
Flat/unit/apart.	64 (3.2)	2030 (62.8)	3231	0.92 (0.85–1.00)
Mobile home/other	11 (3.9)	281 (58.3)	482	0.86 (0.71–1.04)
Carer status				
No	296 (1.6)	18427 (67.8)	27178	Ref
Yes	51 (2.2)	2303 (63.6)	3621	0.88 (0.82–0.95)
Self-rated health				
Excellent	37 (1.0)	3893 (74.6)	5219	Ref
Very good	116 (1.4)	8420 (70.9)	11876	0.88 (0.82–0.95)
Good	133 (2.1)	6436 (64.5)	9984	0.74 (0.68–0.80)
Fair	52 (3.0)	1729 (54.5)	3170	0.58 (0.52–0.65)
Poor	9 (3.6)	252 (45.8)	550	0.53 (0.43–0.65)
Functional limitation (fl)				
No fl	163 (1.0)	15710 (69.8)	22510	Ref
Slight fl	95 (3.4)	2802 (65.8)	4261	1.04 (0.96–1.12)
Moderate fl	35 (3.1)	1119 (59.2)	1891	0.94 (0.85–1.05)
Significant fl	35 (5.1)	686 (54.1)	1267	0.81 (0.71–0.92)
Severe fl	19 (4.6)	413 (47.5)	870	0.72 (0.61–0.84)
DSSI in quartiles				
1	179 (2.1)	8383 (69.6)	12039	Ref
2	62 (1.2)	5063 (68.2)	7428	0.98 (0.92–1.05)
3	50 (1.4)	3611 (66.3)	5447	0.96 (0.90–1.03)
4	56 (1.5)	3673 (62.4)	5885	0.89 (0.83–0.96)

The estimated probability of responding, or the propensity score, derived from the multivariable logistic regression model described above was used to obtain a probability weight for each individual. For the responders, this weight is simply the inverse of the propensity score, known as the inverse probability weighting (IPW) [6]. The goal of this method is to weight individuals with lower propensities for response more heavily than those with higher propensities. The effect is that responders represent themselves and nonresponders who have similar characteristics in order to offset for the missing responses. The IPW approach is valid under a MAR assumption. That is, the probability of responding to the follow-up questionnaire is independent of the outcome, conditional on the set of observed covariates used to compute the weights. This is a strong assumption that asserts, given the observed covariates, those who do not respond behave in similar ways to those who do respond. This assumption is impossible to verify in practice without collecting data on the nonresponders.

For modelling the outcome variable, transition to residential aged care facility, univariate Chi-squared analysis was firstly used to identify statistically significant associations for each variable described above. Those variables with significant associations with the transition were included in a multivariable logistic regression model to further test associations. Possible first-order interaction terms between the following variables were also considered: sex with income, marital status, work status and age group; age group with physical function, health status, country of birth and language spoken at home. Interaction terms were first added to the main effects model one at a time and those with a *p*-value > 0.05 were dropped from the model. Then we sequentially add those interaction terms with significant *p*-values and after inclusion of main effects and other interaction terms, those with *p*-value > 0.05 were dropped to obtain the final model. This complete case analysis was repeated with survey commands that allowed for weighting responders according to their propensity scored derived weights (“complete case with IPW”). Note that other variable selection methods due to shrinkage, such as the Least Absolute Shrinkage and Selection Operator (LASSO) can be applied. The complete case analysis and the complete case with IPW were performed using SAS version 9.3.

If the MAR assumption was violated, then IPW adjustment may not necessarily remove all nonresponse bias. This leads to the missingness mechanism known as “not missing at random” (NMAR) or informative missingness, where the probability of a missing value depends on the value of the variable that is missing. In this case, the missing data mechanism must be specified by the researcher and incorporated into the model in order to obtain unbiased parameter estimates. However, available data contains no information about what would be an appropriate model for the missing data and statistical inference is very sensitive to the choice of such model. This makes sensitivity analysis essential for investigating possible violations of the MAR assumption and exploring the robustness of the study conclusions to increasingly extreme departures from the MAR mechanism ([24, 30–32]).

In this paper, we adopt a selection model approach for NMAR, which consists of two sub-models: one specifies the relationship between the covariates and the outcome of interest and the other represents the missing data process, which is dependent not only on observed covariates, but also the outcome. More specifically, we assume a standard logistic regression for the transition to residential aged care:

1 $$ \mathrm{logit}\left( P\left({y}_i=1\right)\right)={b}_0+{\displaystyle \sum_{j=1}^k}{b}_j{x}_{j i}, $$

where *y*_*i*_ is the outcome and *x*_*ji*_ is the *j*th baseline covariate for subject *i*. Potential covariates for the outcome, as well as those that may be predictors for nonresponse are detailed in Additional file 1. We then specify a logistic model for missingness as follows:

2 $$ \mathrm{logit}\left( P\left({m}_i=1\right)\right)={\theta}_0+{\displaystyle \sum_{s=1}^l}{\theta}_s{x}_{s i}+\lambda {y}_i, $$

where *m*_*i*_ is a nonresponse indicator taking a value of 1 if the *i* th individual did not respond to the follow-up questionnaire, 0 otherwise. Other viable modelling frameworks for analysing data with informative missingness include pattern mixture models [33] and shared parameter models [34].

In the above selection model we assume a linear relationship between the logit of the probability of nonresponse and the outcome. Different values of the parameter *λ* posit different assumptions on how strongly the likelihood of nonresponse depends on the outcome. When *λ* = 0, we have the MAR case where the probability of nonresponse only depends on observed covariates. This case corresponds exactly to the logistic regression model used to construct IPW weights and it further shows the selection model is an appealing choice as it relates to the propensity score method but model parameter are estimated jointly rather than in a two-stage process. More generally, the parameter *λ* is interpreted as the log odds ratio of nonresponse for those who had a dwelling-type change, conditional on all other covariates included in the model. We make the assumption that *λ* is nonnegative, that is, the likelihood of nonresponse is higher for those who had a dwelling-type change. This is a plausible assumption since change dwelling is often associated with family-type events such as marriage or birth and work transitions [35]. Thus, those who had dwelling-type change are more difficult to track in a longitudinal study as well as other sorts of changes in life course, making them less likely to respond to the follow-up survey [36]. In implementing the selection model, we repeat the analysis for a range of values of *λ* and examine the sensitivity of the estimated regression coefficients in the outcome equation across these values. The values we set for *λ* are (0, 1, 2, 3). More specifically, these values imply that the odds ratio of nonresponse for individuals with a dwelling-type change (which is transition into aged care facilities) is between 1 and 20 ([36, 37]). Note that in practice, one could also assign a mildly informative prior distribution to *λ* and estimate it jointly with other model parameters.

A full Bayesian probability modelling approach using Markov chain Monte Carlo (MCMC) was used for the selection model, as it was shown that the Bayesian modelling approach provides a flexible way to incorporate different assumptions on the missing data mechanism and enables coherent model estimation ([24, 38, 39]). We ran the selection model in the WinBUGS software [38, 40] for 15,000 iterations including 5000 for burn-in. Vague *N*(0, 1000) prior distributions were assigned to intercept parameters *b*_0_ and *θ*_0_ and all coefficients *b*_*j*_ and *θ*_*s*_ in equations (1) and (2). Visual inspection of trace plots and autocorrelation plots of MCMC iterations was satisfactory suggesting that all runs achieved convergence.

## Results

Table 1 presents the distribution of demographic and other characteristics at baseline including work status, dwelling type, carer status, self-reported health conditions, physical function limitation and social connectedness among responders and nonresponders who were not residing in a residential aged care facility at baseline. After removing those with missing values in any baseline covariate listed in Additional file 1, 67.3% of individuals responded to the follow-up survey. Odds ratios (OR) and 95% confidence interval (CI) estimated from the multivariable logistic regression model for deriving propensity score weights for responders and nonresponders are also presented in Table 1. Table 1 also provides the percentages of subjects within each category who had transitioned to an aged facility at follow-up.

The results of the propensity score modelling show that individuals with the following characteristics have a higher probability of responding to the follow-up survey as compared with each reference category: female, in 55-74 age category, having higher educational qualifications, being married or in a de facto relationship and having higher household income. Conversely, those who at baseline survey worked full-time, had poor self-rated health, had significant to severe functional limitation, poor social connectedness, were a carer or were born outside Australia are more likely to be nonresponders at follow-up.

The mean propensity score for responders was 0.31 with a standard deviation of 0.11. The average weight given to each responder was 3.65 (range: 1.22 – 8.54). The C statistic or the area under the ROC curve for the model was equal to 0.65.

Table 2 shows ORs and 95% CIs for the complete case analysis (with and without IPW) and for the selection model with different values of *λ* for the association between transition and various baseline characteristics. For the complete case analysis without any adjustment for nonresponse, the likelihood of making the transition is significantly greater for individuals who were over 55 years of age and who had slight, significant or severe physical functional limitation. On the other hand, those who worked full time or who lived in a house/house on farm were significantly less likely to transition into residential aged care facility between baseline and follow-up.

**Table 2:** Multivariate logistic regression analysis of characteristics associated with changed dwelling type between baseline and follow-up survey

Baseline characteristics	Complete case	Complete case with IPW	Selection model with *λ* = 0
Gender			
Male	Ref	Ref	Ref
Female	1.156 (0.92–1.46)	1.102 (0.86–1.41)	1.158 (0.93–1.45)
Age (yrs)			
45–54	Ref	Ref	Ref
55–64	**5.369 (2.29–12.60)**	**6.395 (2.62–15.60)**	**5.371 (2.47–11.55)**
65–74	**16.301 (6.83–38.93)**	**21.294 (8.53–53.17)**	**16.445 (7.30–36.31)**
75+	**43.021 (17.80–104.00)**	**58.047 (23.11–145.82)**	**43.598 (19.01–97.32)**
Marital status			
Single	Ref	Ref	Ref
Married/de facto	0.806 (0.49–1.33)	0.773 (0.45–1.34)	0.816 (0.49–1.41)
Widowed/divorced/separated	0.608 (0.36–1.04)	0.613 (0.35–1.09)	0.613 (0.36–1.09)
Work status			
FT/self-employed	Ref	Ref	Ref
PT	**2.235 (1.20–4.16)**	**2.455 (1.29–4.68)**	**2.189 (1.15–4.15)**
Fully retired	**3.710 (2.25–6.13)**	**3.816 (2.24–6.49)**	**3.717 (2.20–6.16)**
Partially retired	**2.690 (1.32–5.49)**	**2.409 (1.15–5.06)**	**2.591 (1.22–5.28)**
Disabled/sick	1.754 (0.51–6.05)	2.400 (0.64–9.03)	1.484 (0.32–4.90)
Unemployed/look after home/study/unpaid	**2.386 (1.18–4.83)**	**2.604 (1.25–5.43)**	**2.308 (1.11–4.60)**
Dwelling type			
House/house on farm	Ref	Ref	Ref
Flat/unit/apart.	**1.522 (1.13–2.04)**	**1.485 (1.09–2.03)**	**1.511 (1.12–2.02)**
Mobile home/other	**1.929 (1.02–3.66)**	1.797 (0.94–3.44)	1.866 (0.93–3.51)
Functional limitation (fl)			
No fl	Ref	Ref	Ref
Slight fl	**1.702 (1.30–2.23)**	**1.704 (1.29–2.26)**	**1.703 (1.30–2.24)**
Moderate fl	1.301 (0.88–1.91)	1.318 (0.87–1.99)	1.287 (0.86–1.90)
Significant fl	**2.060 (1.39–3.06)**	**2.120 (1.40–3.22)**	**2.040 (1.37–3.03)**
Severe fl	**1.917 (1.15–3.20)**	**1.934 (1.12–3.35)**	**1.885 (1.10–3.10)**

Bold font indicates statistically significant results at 5% level

Table 2 reveals some interesting findings. First, results based on the propensity score analysis were very similar to those based on the naïve complete case analysis. Specifically, there were only small changes in estimated ORs and no change in conclusions regarding associations with the transition of interest. The one exception was in the case with IPW, the likelihood of transition changed from borderline to non-significant for those living in mobile home/other as compared to those who lived in a house at baseline. In general, CIs were generally slightly wider for the propensity score-based analysis, due to the variability in the derived weights [41].

Second, Table 2 suggests that results based on the Bayesian selection modelling with *λ* = 0, were very close to the IPW and complete case analyses. This is to be expected since *λ* = 0 corresponds to MAR, as discussed above.

Finally, Table 2 provides a useful assessment of the potential impact of informative missingness by presenting results based on three different values of with *λ*. The degree of departure from MAR becomes more extreme as *λ* increases. For the most part, results were surprisingly robust, even for large values of *λ*. There were some cases, however, when the estimated ORs and conclusions changed. For example, the odds ratio for comparing the likelihood of transition into aged care facility between those who lived in mobile home/other to those lived in house at baseline became statistically significant under the NMAR assumption. The odds ratio estimates for baseline work status were significantly positive (except for “disable/sick”) in the complete case analysis, models assuming MAR and the selection model with *λ* = 1. However, as *λ* increases, the point estimates for categories “work part-time”, “partially retired” and “unemployed/other” shifted toward the null value and became insignificant when *λ* = 3. In contrast, the estimate for “disabled/sick” shifted away from the null value and the CI no longer included 1 as *λ* increased. Lastly, for physical functional limitation, the only change in conclusion occurred for the category “moderate functional limitation”, where the estimate became significant in NMAR with *λ* ≥ 2.

**Fig. 1:**
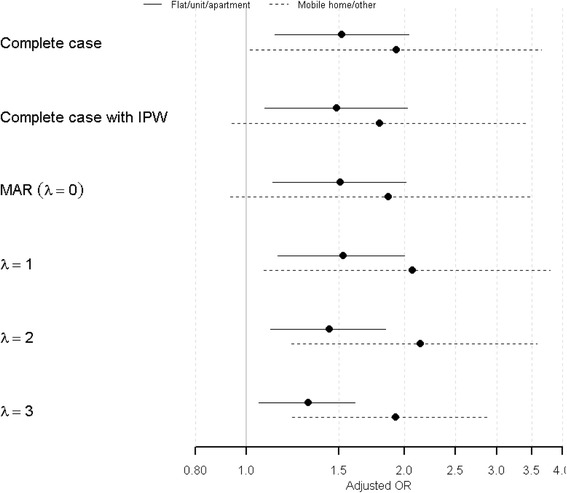
Odds ratio and 95% CI for gender (Ref = “Male”), under the complete case, complete case with IPW and selection model with different values of *λ*

**Fig. 2:**
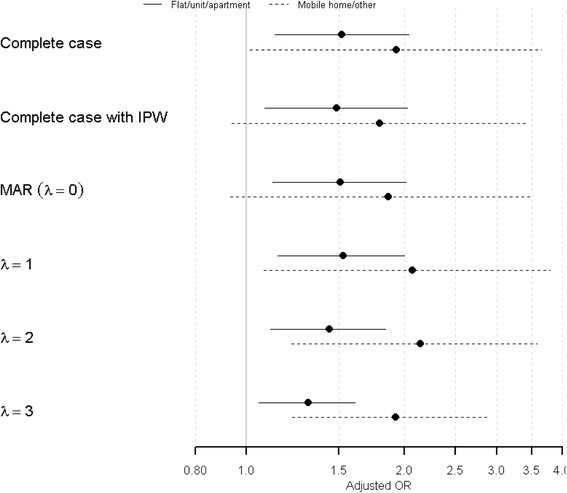
Odds ratio and 95% CI for age group (Ref = “45-54”), under the complete case, complete case with IPW and selection model with different values of *λ*

**Fig. 3:**
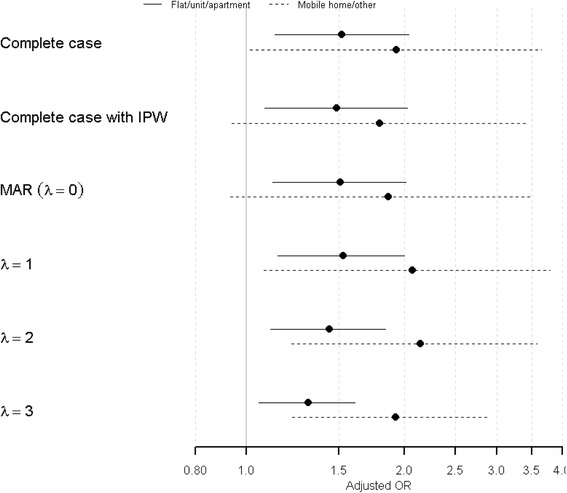
Odds ratio and 95% CI for dwelling type (Ref = “House”), under the complete case, complete case with IPW and selection model with different values of *λ*

Selected forest plots showing how OR estimates and CIs change under different modelling assumptions (i.e. complete case, MAR and NMAR with increasing values of *λ*) are shown in Figs. 1, 2 and 3. These plots clearly demonstrate the overall robustness of our conclusions to the possibility of MAR and NMAR assumptions, though we see some sensitivity of the estimates related to baseline work status. The remaining forest plots are available online as supplementary material (see Additional files 2, 3 and 4).

Combining the results of propensity score modelling in Table 1 and the OR estimates for the outcome variable under different assumptions as shown in Figs. 1, 2 and 3, it became clear that some variables are more sensitive to the underlying missing data mechanism and increasing departure from the MAR assumption. We are then able to classify these variables according to their sensitivity to the missingness assumptions and their strength of relationship with both the response and the outcome variable as shown in the following table.

## Discussion

A major threat to the validity of longitudinal studies is nonresponse, which can potentially affect the magnitude and direction of measures of association and in turn can lead to erroneous conclusions. Using the baseline and follow-up data from the Sax Institute’s 45 and Up Study, we were able to identify a large number of factors associated with response to the follow-up survey in this large cohort. More than 65% of the invited participants from the baseline responded to the follow-up survey.

Characteristics associated with a higher probability of responding to the follow-up questionnaire included: female gender, age categories 55–74, higher educational qualification, married/de facto, worked part time or partially or fully retired and higher household income. Those who were born outside Australia, who spoke a language other than English at home, were a carer, who reported poorer subjective health, who had significant functional limitation and poor social connectedness were less likely to respond to the follow-up survey. There is no statistically significant difference in response by area remoteness and baseline dwelling type. Generally speaking, our findings on the characteristics associated with higher probability of response are in accordance with many previous studies [42, 43].

After assessing the factors associated with response to the follow-up survey, we then evaluated the extent to which nonresponse could impact the estimation of change in dwelling type, in particular, transition into a residential aged care facility, between baseline and follow-up survey. To determine if estimates obtained from fitting a logistic model to complete cases only were affected by nonresponse, the first approach was to use propensity score to weigh each follow-up responder. The idea behind the weighting is that an individual with a low predicted propensity for response, who actually responded, will represent a larger group of individuals who did not respond. This approach has been used in many studies ([8, 44]) to assess nonresponse bias in cohort studies. Our results showed that adjusting for nonresponse by the means of using IPW had very little impact on the estimates of dwelling-type change related to various baseline characteristics.

The underlying assumption for using propensity score derived weights to adjust for nonresponse is MAR, which means the probability of nonresponse is independent of any unobserved data, conditional on the observed data. The MAR assumption is commonly used in the literature on missing data methods and an often used justification for MAR assumption is the availability of rich baseline information for characterising both responders and nonresponders [45].

The use of a Bayesian selection model allows us to further assess the robustness of parameter estimates and conclusions when we have reasons to believe the missingness mechanism is NMAR or informative. In implementing the Bayesian selection model, we repeated our analysis over a range of fixed values of parameter *λ*, which controls the degree of departure from MAR assumption. [24] showed that it is not possible to estimate the parameter *λ* and that the observed likelihood is nonidentifiable in the case of logistic regression with informatively missing outcomes. Hence we adopted a sensitivity analysis approach and repeated the analysis for various fixed values of *λ*, which is equivalent to assigning a fixed point prior. The results from the selection model indicate that nonignorable nonresponse did not substantially affect estimates and conclusions regarding statistical significance of variables that were associated with transition into residential aged care facility for *λ* values which are not too extreme.

Table 3 shows that variables that are strong predictors for both the outcome and nonresponse are those affected to a greater extent by increasing departure from the MAR assumption. For instance, gender, marital status and baseline dwelling-type variables are quite robust to different missingness assumptions since they are weak predictors for either nonresponse or the outcome. Note that even with little change in magnitude, the estimate for those living in mobile home/other at baseline became significant in the NMAR analysis.

**Table 3:** Classification of variables according to their strength of relationship with nonresponse and outcome

Predictor of nonresponse	Predictor of the outcome	Sensitivity to missingness mechanism	Variables
Strong	Strong	Yes	Age; work status
Strong	Weak	No	Gender; marital status
Weak	Strong	No	Dwelling type; physical functional limitation
Weak	Weak	No	-

Due to low counts in this category, this result is of borderline insignificance under MAR assumption despite large estimated effect combined with wide CIs. A similar explanation is given for the category “moderate limitation” in the functional limitation variable, where small change in estimate caused the conclusion to change. On the other hand, age and work status as strong predictors for both nonresponse and the outcome, have their estimates varied substantially with increasing *λ* values. However, even with a large change in the estimates of the age variable, conclusions remain unchanged due to large magnitude of the estimated ORs and tight CIs.

This high level of robustness in estimates is consistent with the findings in Wang et al. [24]. Using a simpler model with one binary covariate, they derived exact expressions for the bias in estimates when using complete cases only. From that, it was shown that if the covariate is a weak predictor for the response missingness, then the bias of its regression coefficient in the outcome equation diminishes.

In practice, we suggest caution when interpreting results for variables that are strong predictors for both nonresponse and outcome, as their estimates are sensitive to varying missingness assumptions and departure from the MAR assumption. On the other hand, small changes in estimates of variables which are not strong predictors of nonresponse and/or outcome could still result in change in conclusions when they are of borderline significance with wide CIs. Furthermore, change in conclusion usually occurs with large values of *λ*, which could be too extreme or scientifically implausible in a particular context.

Results based on our Bayesian selection modelling revealed a surprising level of robustness in terms of estimated ORs and associated CIs. A helpful explanation for this may be obtained through drawing an analogy with the familiar case control setting. There, it is well known that oversampling cases does not affect estimated covariate effects, only the estimated intercept in a logistic regression analysis of a binary outcome. The presence of informative missingness in an epidemiological study means that responders and nonresponders are differentially represented in the study sample just as in a case-control study. Estimated ORs will only be affected if the missingness mechanism also depends on the same covariates that we wish to correlate with the outcome of interest. This explains what we have observed in Table 2 and in the Figures, namely that estimated ORs are sensitive to informative missingness only for covariates that are strong predictors of missingness as well.

Even though our results showed odds ratio estimates of the covariates and their CIs are generally robust to different assumptions on the missingness mechanism, the intercept estimate is affected, just like in a case-control setting as mentioned above. Hence, any computation that requires the full set of parameter estimates such as calculating predicted probabilities would be impacted by the different assumptions.

There are several limitations in this study. First, we have assumed a linear pattern of missingness in the selection model; it may be worthwhile to explore alternative specifications of the model of missingness. Second, it is possible that there are some unmeasured factors associated with the outcome of interest and/or nonresponse which were not captured. However, since a large number of variables were collected at baseline, the likelihood of uncaptured confounders is low. Sensitivity analyses could be performed to assess how strong the effects of the unobserved confounder on the exposure and/or the outcome would have to be in order to overturn a study conclusion [46]. Third, in our application we did not distinguish between different types of nonresponse. For example, reasons for nonresponse could include refusal or inability to be contacted. This can be accounted for by extending the model for nonresponse by using multiple missingness indicators for each cause. Also, we restrict our analysis to individuals with fully observed covariates at baseline, those with missing values in any baseline covariate could be incorporated by using methods such as multiple imputation. Lastly, we conducted the sensitivity analysis for a range of *λ* values which we assume to be plausible for specifying the probability of nonresponse for individuals with and without transition into an aged care facility. Ideally we want to find strong scientific evidence to support the use of particular values of *λ* or to elicit expert knowledge about the odds of nonresponse for different dwelling change outcomes.

## Conclusions

To the best of our knowledge, ours is the first study to investigate the impact of nonresponse in the 45 and Up Study cohort. We identified several baseline characteristics that were associated with high probability of nonresponse and many of them were commonly cited in the literature. By comparing odds ratio estimates for transition into residential aged care facility using complete case analysis, inverse probability weighted method assuming MAR and Bayesian selection model with the special case of MAR, our results showed in the context of examining factors associated with transition into aged care facilities, nonresponse did not result in substantial bias and certainly did not alter the interpretation of the results in general. We further examined the robustness of the conclusions under the NMAR assumption with varying degree of departure from MAR and showed the conclusions were still largely consistent for plausible values of *λ*.

It is important to note that the MAR and NMAR classification is unverifiable from the data under analysis and remains an assumption, hence making sensitivity analysis essential. Lastly, we describe in detail situations where the NMAR assumption and different values of *λ* are likely to affect parameter estimates and corresponding conclusions. We recommend caution when interpreting results especially where variables are strong predictors for both nonresponse and the outcome, since their estimates and conclusions can be sensitive to the underlying missing data mechanism and varying degree of departure from the MAR assumption. Use of a Bayesian selection model is recommended as a useful strategy for assessing potential sensitivity of results to missing data.

## Additional files

Additional file 1: Provides a table of description of selected variables collected in the 45 and Up Study baseline survey. (DOCX 28 kb)

Additional file 2: Provides a forest plot of OR estimates and CIs under different modelling assumptions for the variable “work status”. (EMF 135 kb)

Additional file 3: Provides a forest plot of OR estimates and CIs under different modelling assumptions for the variable “marital statuts”. (EMF 74 kb)

Additional file 4: Provides a forest plot of OR estimates and CIs under different modelling assumptions for the variable “functional limitation”. (EMF 120 kb)
