# Supplementary material for: On the impact of nonresponse in logistic regression: application to the 45 and Up study
Source: BMC Med Res Methodol. 2017 May 8;17:80. doi: 10.1186/s12874-017-0355-z (PMC5422892; doi:10.1186/s12874-017-0355-z)
Supplement: Supplementary file 1 — Provides a table of description of selected variables collected in the 45 and Up Study baseline survey. (DOCX 28 kb) [file 12874_2017_355_MOESM1_ESM.docx]

**Additional file 1**

**Table Description of selected variables in the 45 and Up Study baseline survey**

| Name | Description | Details |
| --- | --- | --- |
| gender | Gender | 2 levels : 1 = male, 2 = female |
| age | baseline age group | 5 levels: 45-54; 55-64; 65-74; 75-84; 85+ |
| qual | baseline qualification | 6 levels: no qualification (‘none’); School or intermediate certificate (‘Year 10’); Higher school or leaving certificate (‘Year 12’); trade or apprenticeship (‘trade’); Certificate or diploma (‘Cert.’); University degree or higher (‘Tertiary’) |
| cob | country of birth | 11 levels: Australia; North West Europe (‘NW Europe’); Southern & Eastern Europe (‘S & E Europe’); North Africa & Middle East (‘Middle East’); South East Asia (‘SE Asia’); North East Asia (‘NE Asia’); Southern & Central Asia (‘S & Central Asia’); America; Sub Saharan Africa; Oceania & Antarctica (not Australia) (‘Oceania’) |
| otherlang | speak language other than English at home | 1 = yes; 0 = no |
| marital | marital status at baseline | 6 levels: single; married; de facto; widowed; divorced; separated |
| work | work status at baseline | 7 levels: work full time or self-employed (‘FT’/self-employed’); work part time (‘PT’); fully retired; partially retired; disabled/sick; look after home/study/unpaid work; unemployed |
| income | income category | 5 levels: <$20,000; $20,000-$40,000; $40,000-60,000; $60,000-$80,000; >$80,000 |
| house | dwelling type | 8 levels: House; Flat/unit/apartment (‘Flat/unit/apart.’); house on farm; retirement village; nursing home; hostel for the aged; mobile home; other |
| carer | carer status | 1 = carer; 0 = not a carer |
| health | self-rated health | 5 levels |
| SF36 | physical function limitation (fl) | assessed using the RAND 36-Item health Survey; 5 levels: no fl; slight fl; moderate fl; significant fl; severe fl |
| soc | social connectedness | assessed using the Duke Social Support Index (DSSI) subscale; larger number means worse social connectedness; 4 levels |
